# Development of a Mineral Binder for Wood Wool Acoustic Panels with a Reduced Carbon Footprint

**DOI:** 10.3390/ma18214999

**Published:** 2025-11-01

**Authors:** Aleksandrs Korjakins, Genadijs Sahmenko, Ina Pundiene, Jolanta Pranckevicienė, Vjaceslavs Lapkovskis

**Affiliations:** 1 Institute of Sustainable Building Materials and Engineering Systems, Faculty of Civil and Mechanical Engineering, Riga Technical University, Kipsalas 6A, LV-1048 Riga, Latvia; aleksandrs.korjakins@rtu.lv (A.K.); genadijs.sahmenko@rtu.lv (G.S.); 2 Laboratory of Concrete Technologies, Institute of Building Materials, Vilnius Gediminas Technical University, Sauletekio av. 11, LT-10223 Vilnius, Lithuania; jolanta.pranckeviciene@vilniustech.lt

**Keywords:** binder, CO_2_, wood wool, composite, life cycle assessment

## Abstract

The construction industry’s reliance on Portland cement (PC) significantly contributes to global CO_2_ emissions, driving the search for sustainable binder alternatives. This study develops and evaluates novel mineral binder systems for wood wool acoustic panels with a reduced carbon footprint. Alternative binders, including calcium aluminate cement (CAC), magnesium oxychloride cement (MOC), and gypsum–cement–pozzolan (GCP) hybrids, were combined with additives such as metakaolin and liquid glass. Mechanical testing demonstrated that 20–30% metakaolin and liquid glass composites achieved flexural strengths of up to 2.65 MPa and densities above 490 kg/m^3^. The GCP system showed synergistic improvements in flexural and compressive strengths by nearly 50%, along with enhanced dimensional stability and water resistance. Life cycle assessment indicated substantial CO_2_ emission increases, particularly for the MOC and CAC formulations, compared to conventional Portland cement-based panels. The carbon footprint of the binder system consisting of GCP is approximately 5.644 kg of CO_2_ equivalent per functional unit compared to magnesium chloride binder systems, which reach up to 10.84 kg CO_2_ eq., and white Portland cement systems, which are around 6.19 kg CO_2_ eq. The three-component GCP binder system offers the best balance of mechanical performance and minimised environmental impact. Key raw material contributors to the ecological load are cement (various types), MgO, MgCl_2_, and metakaolin, highlighting the importance of optimising binder formulations to reduce carbon emissions. The GCP system, in particular, demonstrates unprecedented synergistic improvements in flexural and compressive strengths, dimensional stability, and water resistance while minimising CO_2_ emissions. Current work sets a new benchmark for sustainable building materials by offering an eco-innovative pathway towards low-carbon, high-performance wood wool acoustic panels, aligning with global decarbonisation goals.

## 1. Introduction

Wood wool acoustic panels are an emerging class of sustainable building materials that have garnered attention due to their favourable acoustic performance, environmental benefits, and multifunctional properties. These panels, often referred to as wood wool cement boards, combine wood wool, typically obtained as excelsior or fine wood fibres, with Portland cement and water. The resulting composite exhibits a porous cellular structure, which is fundamental to its sound-absorption capabilities [1,2].

The acoustic performance of wood wool cement panels is primarily a function of their microstructure [3]. This porous structure facilitates the dissipation of acoustic energy through multiple internal reflections and frictional losses, thereby enhancing the panels’ efficacy in reducing noise.

In addition to their acoustic properties, wood wool acoustic panels offer desirable mechanical characteristics and environmental performance. Studies have shown that the bonding between wood fibres and the cementitious matrix yields a material with good structural stability and fire resistance, making these panels an attractive alternative to conventional mineral wools and synthetic absorbers [4]. The hygroscopic behaviour of the wood fibres, particularly when derived from spruce, contributes to dimensional stability and improved sound damping under fluctuating moisture conditions [5,6]. The classification and mechanistic understanding of wood-based composites elucidate the balance between sound absorption, porosity, and mechanical strength densification. Wood wool acoustic panels incorporating Portland cement are fabricated by combining wood wool—typically derived from wood fibres of various dimensions—with Portland cement; water; and, in some cases, additives that enhance workability and interfacial bonding [7]. This formulation produces a lightweight, highly porous composite that imparts the fire-retardant properties of Portland cement, owing to a hydrate layer that acts as a barrier against ignition [8]. Acoustic performance in wood wool cement panels is primarily achieved by creating an open cellular structure. The crucial role of the composite’s porosity and fibre distribution is reflected in the mechanical properties of wood–cement composites [1]. The hierarchical structure resulting from the interpenetration of fibres within the cementitious network imparts rigidity while maintaining a degree of flexibility, which is beneficial in applications requiring both load-bearing capacity and acoustic damping [9]. Additionally, wood wool cement boards are noted for their low density and high thermal insulation properties, making them suitable for interior cladding or partition elements in environments where thermal and acoustic comfort are priorities [10].

However, traditional acoustic panels often utilise petroleum-based or non-renewable resources, contributing to environmental degradation through high embodied energy and waste generation. Mineral wool, which encompasses both rock wool (also known as stone wool) and glass wool, serves as a crucial component in the field of acoustic insulation [11]. Its fibrous structure provides high acoustic absorption capabilities alongside important thermal insulation properties, making it integral in construction and engineering applications. Miškinis et al. [12] demonstrated that fibrous materials exhibit high absorption performance across various frequency ranges while also providing beneficial mechanical strength and thermal insulation.

Recent studies indicate that recycling waste materials and incorporating bio-based constituents, such as cellulose-nanocrystal-modified luffa structures, cork granulates, tree bark [13], and recycled tea bags, can yield acoustic panels with competitive sound absorption properties while significantly reducing their environmental footprint [3,14,15,16,17,18,19]. The integration of natural fibres and bio-based composites into acoustic panel design presents an eco-innovative solution that utilises renewable resources.

The integration of Portland cement ensures that the panels resist moisture-induced degradation and display improved fire resistance, a critical attribute derived from the cement’s barrier effect—as evidenced by performance evaluations conducted on cement-bonded materials [4]. This fire-retardant property, coupled with the natural biodegradability and recyclability of wood wool, underscores the environmental benefits of these composites. Integrating fly ash and ground granulated blast-furnace slag, pozzolans can lead to a reduction in the demand for Portland cement, thereby decreasing CO_2_ emissions [20,21,22] because up to 10% of worldwide anthropogenic CO_2_ emissions are attributed to cement production, highlighting its role as a major contributor to climate change [20,23,24,25].

The design of sustainable construction practices involves transitioning towards geopolymer concrete, which entirely replaces Portland cement [4,26,27], as well as eco-innovative binders, such as magnesium oxychloride cement (MOC), magnesium phosphate cement (MPC), calcium aluminate cement (CAC), and blended systems incorporating pozzolans and gypsum. These alternatives reduce CO_2_ emissions during production by utilising lower calcination temperatures and alternative chemistry and offer enhanced properties such as rapid curing, improved fire resistance, moisture durability, and compatibility with renewable fibrous materials like wood particles. These alternative binders also enable the use of renewable fibrous materials, such as wood wool, which enhance acoustic absorption through controlled porosity and fibre hybridisation techniques.

The transition from PC to these sustainable alternatives forms the foundation for developing next-generation composite materials designed for acoustic and structural applications in the built environment. Alternative binders, such as CAC and MOC, exhibit lower embodied carbon, resulting in a more sustainable lifecycle compared to PC. This supports global efforts to decarbonise the construction industry. Enhanced hydration and curing performance can be achieved by using CAC; MOC; and hybrid three-component binder systems that combine gypsum, cement, and pozzolans [28]. This encourages the development of rapid-setting binders and enables shorter production cycles and faster formwork removal, thereby improving manufacturing efficiency and reducing energy consumption. CAC’s excellent sulphate resistance and MOC’s fire retardancy and moisture resistance are crucial for long-term performance in diverse building environments.

Emphasising environmental sustainability, resource efficiency, and superior performance, these innovations represent critical steps toward mitigating the construction industry’s impact on climate change and advancing circular economy principles.

## 2. Materials and Methods

### 2.1. Raw Materials

Raw materials used: wood wool, Portland cement, metakaolin, liquid glass, calcium aluminate cement, Magnesium oxide, Magnesium chloride, gypsum, water.

The wood wool, made from pine, was used to create an acoustic wood wool panel with a mineral binder. The wood wool was produced by the company Stiga RM Ltd. (Tukums, Latvia). The sizes of the wood wool are presented in Table 1. Measurements have been made for 3 g of wood fibres.

White PC CEM I 52.5 R is the binder base for developing wood wool boards. This cement has improved mechanical properties in comparison with ordinary PC and its decorative properties. It provides mechanical strength and durability essential for structural stability. Its production, however, is associated with high CO_2_ emissions, motivating the search for alternative binders. In contrast to ordinary cement, white cement is characterised by very low contents of transition metal oxides (e.g., iron and manganese), which results in a brilliant white colour and higher purity. The composition of white cement is presented in Table 2.

Metakaolin (MK) (Stikloporas Ltd., Druskininkai, Lithuania) was used as the source of aluminium silicate. MK is obtained via the endothermic reaction of kaolin. The analysis of the MK particle size distribution revealed that 90% of the particles were smaller than 76.0 µm, 50% of the MK particles were smaller than 6.0 µm, and 10% were smaller than 1.3 µm. MK is an aluminosilicate additive used as a partial replacement for OPC to enhance composite properties. The composition of MK is presented in Table 3.

Liquid glass (sodium silicate solution Na_2_O(SiO_2_)·xH_2_O) acts as a supplementary additive, improving composites’ durability and water resistance. It contributes to forming a denser, more cohesive microstructure with enhanced interfacial bonding between wood particles and binder, further increasing flexural strength and reducing porosity. Polyline, Latvia, Riga, produces liquid glass.

We used Calcium aluminate cement (CAC) under the brand name GÓRKAL 70 (Trzebinia, Poland). CAC offers rapid curing and early strength development, facilitating shorter production cycles. It shows excellent sulphate resistance, thermal stability, and fire resistance, making it well suited for particleboards exposed to moist or aggressive environments. It provides higher moisture–drought resistance and retains strength after long water exposure, broadening its applicability in humid conditions. The chemical composition of the CAC is presented in Table 4.

Ordinary PC CEM I 42.5 R serves as the conventional binder base in producing wood wool boards. It provides mechanical strength and durability essential for structural stability. The composition of PC is presented in Table 5.

Magnesium oxide (MgO) is applied to produce magnesium oxide chloride cement (MOC). Magnesium chloride is an inorganic compound with the formula MgCl_2_. It forms hydrates MgCl_2_·6H_2_O. These salts are colourless or white solids that are highly soluble in water. MgO was applied to produce magnesium oxychloride cement (MOC).

Gypsum is a soft sulphate mineral of calcium sulphate dihydrate, with the chemical formula CaSO_4_·2H_2_O. Gypsum was produced by the company Knauf, Riga, Latvia.

### 2.2. Alternative Binder Systems

MOC has garnered attention due to its advantageous properties, including rapid setting and hardening, high strength-to-weight ratio, fire resistance, and overall low thermal conductivity [29,30,31]. Due to these properties and a comparatively low carbon footprint, MOC is an attractive alternative for durable, eco-friendly wood particleboard binders.

The gypsum–cement–pozzolan binder (GCP) ternary system synergistically combines gypsum, PC, and pozzolanic materials. Gypsum imparts rapid initial strength, while pozzolans contribute to long-term strength and improved water resistance; PC offers structural integrity. This three-component binder reduces shrinkage and cracking, enhances dimensional stability, and mitigates the formation of deleterious compounds such as ettringite and thaumasite, especially in sulphate-rich or humid environments. Panels with GCP retain higher strength and moisture resistance than those with gypsum or two-component systems, making GCP a more advantageous choice for specific acoustic panel applications.

Water is a crucial component in the processing of cementitious mixtures, facilitating hydration reactions and improving their workability. Its quantity is optimised to balance mixture consistency and mechanical properties across formulations, including those with alternative binders. Tap water (drinking water) was used for mixing preparation.

### 2.3. Key Properties of Raw Materials for Binder and Binder Systems

These raw materials and binders form the basis for developing eco-innovative wood particleboard composites with balanced mechanical performance, improved durability, enhanced fire safety, and significantly reduced environmental impact compared to traditional PC-based materials. The key properties of raw materials for binders and binder systems are presented in Table 6.

### 2.4. Preparation of the Binder Systems and Wood Wool Panel Samples

#### 2.4.1. Elaboration of the Mixtures

A base reference formulation was a commercial version of a wood wool panel prepared using ordinary PC (CEM I 42.5 R), liquid glass, and wood wool. This formulation serves as a benchmark for comparing the performance of alternative binders.

The wood wool is produced from pine wood.The amount of wood wool ranges from 1200 to 2100 g/m^2^.

The ratios between wood wool (WW) and mineral binder (MB) were in the range MB/WW = 2.93 ÷ 4.63 by weight.

Alternative binder formulation variations include different precursors mentioned above in various ratios. The considered precursors (marked with the symbol ✓) are presented in Table 7.

- ASB—Partial replacement of white PC (CEM I 52.5 R) with metakaolin at 0%, 5%, 10%, 20%, and 30% with different PC–wood wool ratios to evaluate mechanical and physical enhancements. The composition of the binder consists of PC, metakaolin, liquid glass, and water.
- ASB-2—Partial replacement of PC with metakaolin at levels of 5% to 30% with a treatment mix of PC and metakaolin in the disintegrators, with a rotation speed of 2000 rpm, to evaluate mechanical and physical enhancements. The binder’s composition consists of PC with metakaolin, liquid glass, and water.
- ASB-5—Partial replacement of PC with metakaolin at levels of 5% to 30% with a treatment mix of PC and metakaolin in the disintegrators with a rotation speed of 5000 rpm to evaluate mechanical and physical enhancements. The binder’s composition consists of PC with metakaolin, liquid glass, and water.
- CACB—CAC replaces PC in the composition. We use different CAC–wood wool and water–CAC ratios for increased hardening speed to evaluate mechanical and physical enhancements. The binder’s composition consists of CAC with a different water–CAC ratio, liquid glass, and water.
- MOSB—Magnesium oxychloride cement (MOC) for moisture performance. The binder’s composition consists of magnesium oxide and magnesium chloride in different MOC–water ratios, as well as water.
- GCP binder system—A three-component mixture of gypsum, PC, and pozzolanic additives, formulated to balance rapid strength gain with long-term durability and dimensional stability. The composition of the binder consists of PC, gypsum, and metakaolin with different ratios; liquid glass; and water

#### 2.4.2. Mixing and Forming

**Figure 1:**
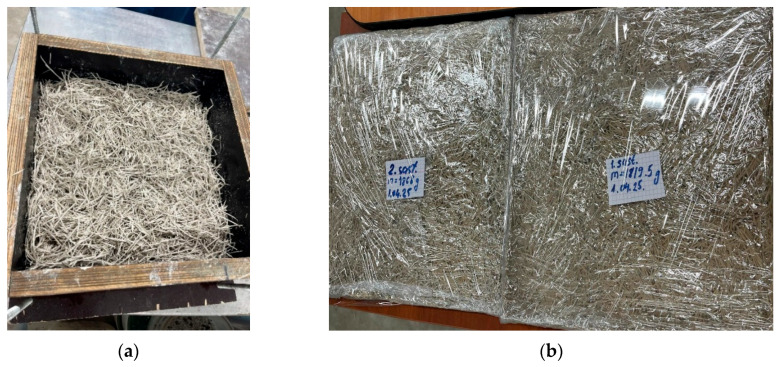
(**a**) Wood wool mixture is placed into pre-prepared formwork. (**b**) Wood wool panels are cured in the film.

The wood wool is moistened with water, binder components are added, and the wood wool is thoroughly mixed with them. Necessary water and additives are then added to form a homogeneous slurry or mixture. The liquid glass additive, if used, is incorporated into the water. The mixture is placed in pre-prepared moulds or formworks (Figure 1a) and compacted under controlled pressures to produce acoustic panels that are 25 mm thick.

Forms are made from humidity-resistant plywood. Pressure and duration are applied based on the findings of a previous investigation. For binders such as MOC, the chemical reaction between magnesium oxide and magnesium chloride occurs during the formation and solidification of the matrix.

#### 2.4.3. Curing and Hardening

The formed wood wool boards are left to cure under controlled temperatures and humidity, allowing the binder to hydrate and develop strength. CAC-based boards cure rapidly, reducing processing time, whereas GCP systems require monitoring to ensure dimensional stability. Typical curing regimes balance initial strength gains (primarily from gypsum or calcium aluminate cement) with long-term strength improvements due to pozzolans. The samples were demoulded after 24 h and then cured under film coverage (Figure 1b) for 7 days.

#### 2.4.4. Testing

The cured particleboards underwent mechanical testing for bending and compression strength in accordance with EN 12089 and EN 826 standards [32,33]. Testing was performed using a Universal Testing System XWN-20 and Controls 3000, (Milan, Italy). Bulk density and thermal properties are evaluated to identify optimum formulations. Thermal conductivity was measured using the FOX600 equipment, produced by TA Instruments (New Castle, DE, USA).

Density was defined after 28 days, when the specimens were cured for 3 weeks at room temperature and 50% humidity. Based on the results, formulations are iteratively adjusted with varying binder ratios, additive treatments (e.g., disintegration intensity for mixtures of metakaolin and Portland cement), and wood wool amounts to maximise performance while minimising environmental impact.

### 2.5. Manufacturing Procedure for Particleboards with Different Binders: Hydration, Mixing, and Homogenisation Steps

The manufacturing process of eco-innovative wood particleboard, combining wood wool fibres and alternative binders with ordinary PC, involves several well-defined stages designed to produce boards with improved structural and environmental performance. The suggested manufacturing process comprises material homogenisation, precise binder mixing, controlled forming and curing conditions, and thorough testing to produce sustainable, high-performance wood wool particleboard composites that contribute to greener building materials.

Wood wool, as a filler, is hydrated to enhance moisture content and ensure stability and adhesion during subsequent processing.

Depending on the formulation, binders such as white PC (CEM I 52.5 R); CAC; magnesium oxychloride cement (MOC); or a three-component system consisting of gypsum, cement, and pozzolan (GCP) are prepared. Additives like metakaolin treated in a disintegrator and liquid glass may be incorporated to improve strength and durability. The hydrated wood wool fibres are mixed with the prepared binder components. A concentrated MgCl_2_ solution is diluted with purified water for magnesium chloride binders and added to the wood particles to distribute magnesium ions evenly. Magnesium oxide powder is then added to the moist wood shavings containing magnesium chloride, and the mixture is thoroughly mixed to form a homogeneous composite material. Dosed water is sprayed to optimise consistency and workability. Proper homogenisation ensures uniform moisture distribution, which is essential for chemical reactions and adhesion within the board matrix.

The resulting homogeneous mixture is placed into pre-prepared moulds or formworks. The mixture is compacted using manual presses. Compression was controlled by moulding forms that influence the thickness of the finished wood wool board.

During curing, chemical reactions occur between the binder components. For example, magnesium oxide reacts with magnesium chloride to form robust magnesium oxychloride cement structures in boards using magnesium chloride as a binder. For gypsum–cement–pozzolan (GCP) systems, gypsum provides rapid initial strength, while pozzolanic materials progressively enhance strength and water resistance over time. CAC-based binders cure rapidly, enabling faster demoulding and reduced construction times. Environmental conditions such as temperature and humidity are controlled to ensure optimal hydration and prevent shrinkage or cracking.

After curing, the particleboards are demoulded. The surface of the particleboard is trimmed to size or laminated for an enhanced appearance. Quality control tests, including mechanical strength, density, thermal conductivity, and visual inspections, are conducted to ensure compliance with specifications.

## 3. Results

### 3.1. Mechanical Properties of Composite Samples

The mechanical property evaluation reveals that white Portland cement and aluminosilicate binders offer enhanced structural strength. At the same time, CAC and magnesium chloride systems provide options for reduced weight or improved thermal characteristics. The results obtained for different samples are presented in Table 8. The results for the benchmark samples are taken from the commercial producer CEWOOD.

The composites developed with white Portland cement consistently surpass the benchmark compressive strength of 0.2 MPa at 10% strain and a flexural strength of 1.3 MPa, achieving flexural strength ranging from 1.31 to 2.00 MPa and compressive strength of up to 0.51 MPa. This indicates a superior load-bearing capacity and stiffness [34], making them well suited for structural applications that require robust mechanical integrity.

Aluminosilicate-based composites exhibit significant increases in flexural strength, ranging from 1.09 to 2.65 MPa, when subjected to high-energy disintegration processes. That suggests that microstructural enhancements via additive processing effectively improve resistance to bending stresses [35,36,37]. However, these gains were sometimes accompanied by slight increases in density, which may affect weight-sensitive applications.

The CACB presented compressive and flexural strengths comparable to white Portland cement, with density values close to the benchmark, indicating a promising balance between mechanical performance and composite weight [38,39]. On the other hand, MOCB exhibited relatively low densities and strength, suggesting potential for lightweight applications but highlighting a need for formulation optimisation to enhance their mechanical robustness.

While the white PC composites showed superior mechanical strength, their thermal resistance was slightly lower than that of other formulations. Conversely, aluminosilicate and GCP-based composites yielded enhanced thermal resistance and comparable mechanical properties. This suggests an inherent trade-off in composite design, where improvements in mechanical strength may coincide with reduced thermal insulation, necessitating optimisation based on the target application requirements.

The high rotation speeds during disintegrator processing (up to 5000 rpm for the ASB-5) appear to activate beneficial modifications in the microstructure, as reflected by improvements in flexural strength and mechanical density properties [35]. That underscores the importance of manufacturing parameters in tailoring composite materials to achieve desired mechanical characteristics.

**Figure 2:**
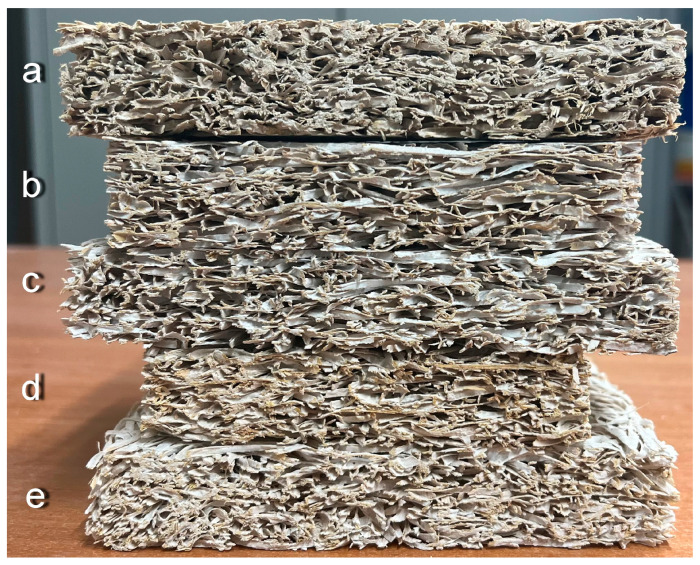
Cross-sections of the wood wool samples: (**a**) MOCB, bulk density: 380.5 kg/m^3^; (**b**) CACB, bulk density: 430.6 kg/m^3^; (**c**) CACB, bulk density: 380.3 kg/m^3^; (**d**) ASB, bulk density: 428.5 kg/m^3^; (**e**) WPC, bulk density: 495.5 kg/m^3^.

**Figure 3:**
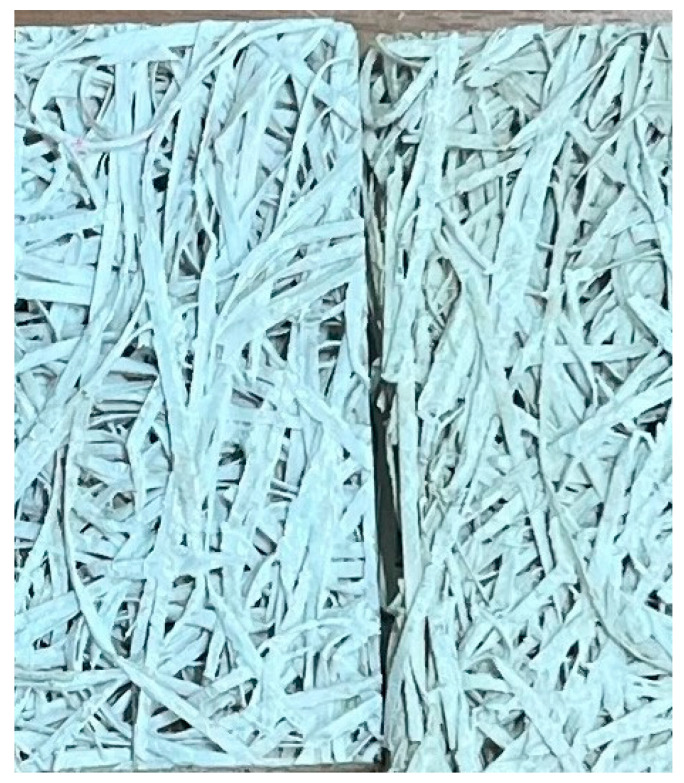
Surface of the wood wool sampled made with CACB and ASB binders.

The cross-sections of the different kinds of samples are presented in Figure 2, where the bulk density varies from 380.5 to 495.5 kg/m^3^. A denser composition structure is observed in the sample with a higher volume density, which may be explained by the denser packing of the wood wool and a greater amount of mineral binder. The surface of the wood wool sampled made with CACB and ASB binders is presented in Figure 3.

The physical-mechanical parameters of optimal wood wool samples with a thickness of 25 mm are presented in Table 9.

The EN 634-2:2007 standard for cement-bonded particleboards [40] requires a density of 1000 kg/m^3^, a bending strength of 9 N/mm^2^, and a modulus of elasticity of 4500 N/mm^2^ for Class 1 boards. Comparing this with the experimental data for optimised wood wool samples of 25 mm thickness reveals some important differences. The experimental boards have densities ranging from approximately 389 to 484 kg/m^3^, which is less than half of the standard requirement of 1000 kg/m^3^. This indicates that the experimental composites are much lighter than the boards specified by EN 634-2. The standard requires a bending strength of 9 N/mm^2^, whereas the experimental boards exhibit flexural strengths ranging from 1.6 MPa to 2.24 MPa. This value is significantly lower than the standard (9 N/mm^2^ = 9 MPa). However, it is important to note that the experimental benchmark listed is ≥1.3 MPa, which the samples exceed. For Class 1 (general cement composites or normal-strength concrete), the standard requires a minimum of 4500 N/mm^2^. Experimentally, the moduli of elasticity range from 2600 to 4480 N/mm^2^, with some boards being close to, but generally lower than, the standard. The standard does not provide a direct compressive strength value here, but the experimental boards have values between 0.31 and 0.6 MPa, exceeding their benchmark of ≥0.3 MPa. The designed experimental boards demonstrate potential as low-density alternatives; however, they currently do not achieve the mechanical performance levels specified for cement-bonded particleboards in EN 634-2:2007.

The incorporation of MK and liquid glass significantly enhances the flexural strength of novel cementitious composites, with optimal compositions, particularly those featuring 20–30% MK, achieving strengths up to 2.65 MPa; this improvement is attributed to MK’s pozzolanic activity and densification effects, further amplified by liquid glass acting as filler, while high-energy disintegration pretreatment of MK contributes to improved reactivity [41]. However, resulting density increases must be carefully balanced against thermal insulation requirements for specific applications.

### 3.2. Effect of Binder Type (CAC, MOC, GCP vs. Portland Cement) on Durability and Thermal Properties

CAC enables rapid early strength gain, achieving high strength within 24 h, much faster than the 28 days required for PC [42]. This rapid curing contributes to faster construction cycles and improved early mechanical performance. CAC also offers excellent resistance to degradation under moist conditions due to its chemical composition, enhancing the panel’s durability in damp environments. Although specific thermal conductivity values are not provided, CAC’s rapid curing and chemical stability imply stable thermal behaviour during panel operation.

MOC cement exhibits excellent adhesion to organic fillers like wood wool, dramatically enhancing flexural and compressive strength, which improves impact resistance and overall panel structural performance [43]. MOC panels also demonstrate superior resistance to moisture, humidity, and mould growth, which provides enhanced durability, especially in wet and fire-prone environments. The superior mechanical strength results are supported by flexural strength values reported in the 1.4 to 2.0 MPa range. MOC has slightly lower thermal resistance compared to PC, which indicates a trade-off—the panels gain mechanical resilience but at the expense of minor insulation performance reduction. Additionally, MOC has a lower carbon footprint than PC, making it an environmentally attractive option.

The GCP binder synergistically combines gypsum, PC, and pozzolanic materials, resulting in rapid initial strength from gypsum and long-term strength from pozzolanic reactions with calcium hydroxide [44]. Compared to two-component systems, this leads to flexural and compressive strength improvements of up to 48–49%. The GCP system also mitigates shrinkage and cracking, significantly improving dimensional stability and moisture-drought resistance ratios. Moreover, it reduces the formation of deleterious compounds like ettringite and thaumasite [45], which enhances durability in sulphate-rich or humid environments. This makes GCP panels particularly well suited for challenging environmental conditions. While exact thermal values are not explicitly outlined for the GCP system, gypsum and pozzolan may influence thermal conductivity, balancing rapid initial strength and long-term stability.

PC is traditionally used for wood wool acoustic panels, with moderate mechanical and thermal performance, but comes with high CO_2_ emissions during production [46]. Thermal resistance and conductivity values for PC-based panels are reported as 0.35 m^2^-K/W and 0.066 W/m-K, respectively. These values serve as benchmarks to assess the alternative binders’ enhancements or trade-offs in thermal and mechanical properties.

### 3.3. Life Cycle Assessment (LCA) Calculations for Wood Wool Cement Panel

#### 3.3.1. Core Components and Structure

All analysed acoustic panels follow a fundamental three-component structure: wood wool as the primary acoustic material, mineral binders for structural integrity, and chemical additives for processing optimisation. Wood wool, typically derived from sustainable forestry operations certified under FSC^®^ or PEFC™ standards, comprises approximately 60% of the panel volume but minimises environmental impact across all formulations. The density of these panels ranges from 387.2 to 423.22 kg/m^3^, with variations primarily driven by binder composition and processing methods.

The fundamental challenge in acoustic panel design lies in striking a balance between performance requirements and environmental considerations. Wood wool provides excellent sound absorption properties due to its fibrous structure and natural porosity, creating air pockets that dampen sound waves. However, the binding system required to provide structural integrity, fire resistance, and durability represents the dominant environmental impact factor.

The seven panel types, considered for LCA, demonstrate diverse approaches to achieving structural bonding while maintaining acoustic performance:

1. The reference cement–wood wool system, which employs standard PC CEM I 42.5 R, representing conventional construction practice with moderate environmental impact;
2. A mineral–wood wool system with aluminosilicate systems, including metakaolin as a pozzolanic additive and white PC (CEM I 52.5 R);
3. A mineral–wood wool system with aluminosilicate systems, including metakaolin as a pozzolanic additive and white PC (CEM I 52.5 R), where PC and MK were treated in the high-speed disintegrator at 2000 rpm;
4. A mineral–wood wool system with magnesium chloride binders represents the most experimental approach, utilising MgO and MgCl2 to create alternative bonding;
5. A mineral–wood wool system with CAC represents a speciality high-performance binder with superior fire resistance;
6. A mineral–wood wool system with a three-component gypsum–cement–pozzolan (GCP) system.

#### 3.3.2. LCA of the Binder System

The seven panel types, considered for LCA, demonstrate diverse approaches to achieving structural bonding while maintaining acoustic performance. The reference cement–wood wool system employs ordinary PC CEM I 42.5 R, representing a conventional construction practice with moderate environmental impact [47]. White PC formulations (CEM I 52.5 R) enhance aesthetic properties through their lighter colour but generate higher CO_2_ emissions due to more energy-intensive production processes.

Aluminosilicate systems incorporate MK as a pozzolanic additive, resulting in hybrid binder systems that may offer enhanced long-term durability. However, these formulations show varying environmental performance depending on whether disintegration treatments are applied during processing [47].

The CAC represents a speciality high-performance binder with superior fire resistance but significant environmental penalties.

The three-component gypsum–cement–pozzolan (GCP) system represents an innovative approach that combines multiple binding mechanisms to optimise performance and sustainability. Magnesium chloride binders represent the most experimental approach, utilising MgO and MgCl_2_ to create alternative bonding chemistry but resulting in the highest environmental impact among all options tested.

#### 3.3.3. Carbon Footprint Analysis

The environmental assessment reveals that binder materials account for 95.3% to 98.2% of total carbon emissions across all panel types, with wood wool contributing only 2–3% to the overall environmental impact.

The reference cement–chip panels achieve the lowest environmental impact at 5.40 kg CO_2_ eq./m^2^, establishing a conventional acoustic panel production baseline. Three-component GCP systems perform similarly at 5.64 kg CO_2_ eq./m^2^, demonstrating that innovative multi-binder approaches can maintain competitive environmental performance while potentially offering enhanced functional properties.

White Portland cement panels generate 6.19 kg CO_2_ eq./m^2^, representing a 14.6% increase over the reference system, primarily due to the higher energy requirements for white cement production. This premium reflects the aesthetic benefits but questions the sustainability of appearance-focused material selections.

Aluminosilicate systems demonstrate variable performance, with disintegrator treatment reducing emissions to 7.11 kg CO_2_ eq./m^2^ compared to 7.92 kg CO_2_ eq./m^2^ without treatment. This suggests that processing modifications can provide meaningful environmental improvements, though both variants remain significantly above baseline performance.

**Figure 4:**
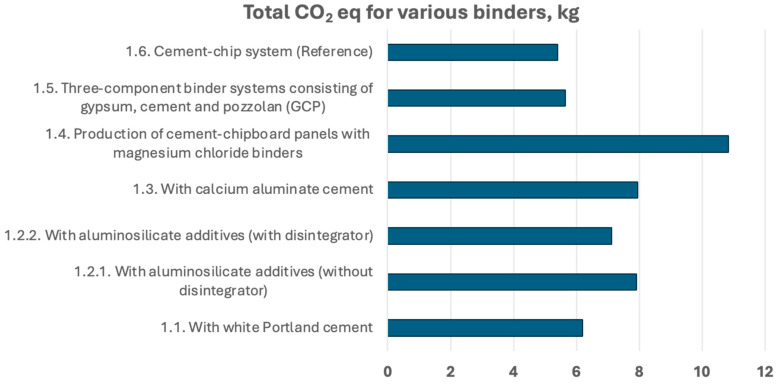
Comparison of CO_2_ emissions across different acoustic panel compositions, showing significant variation from 5.40 to 10.84 kg CO_2_ eq. per m^2^.

CAC-based panels generate 7.94 kg CO_2_ eq./m^2^, the highest among PC-based systems, reflecting the specialised production requirements for this high-performance binder. While offering superior fire resistance and durability, the environmental cost raises questions about cost–benefit optimisation in standard applications (Figure 4).

#### 3.3.4. Worst-Case Environmental Impact

Magnesium chloride binder systems represent the environmental worst-case scenario at 10.84 kg CO_2_ eq./m^2^, contributing 100.8% higher emissions than the reference system. This dramatic increase is attributed to the energy-intensive production of MgO and MgCl2 components, as well as potential transportation impacts associated with these specialised materials. Despite potential advantages in specific applications, the environmental penalty makes these systems unsuitable for mainstream sustainable construction.

## 4. Discussion

### 4.1. Mechanical and Physical Property Enhancement Through Aluminosilicate/MOC/GCP Additives and Processing

Aluminosilicate additives, particularly MK combined with liquid glass, partially replace PC, contributing to significant improvements in composite strength and density. Mechanical activation through high-speed disintegration (up to 5000 rpm) dramatically enhances the pozzolanic activity of these additives by increasing the specific surface area through particle comminution and inducing structural changes in the additives and the cellulose fibres. This leads to denser packing and stronger interfacial bonding within the composite matrix. For instance, formulations containing 20–30% MK and liquid glass processed at 5000 rpm achieved peak flexural strengths of approximately 2.65 MPa and densities exceeding 490 kg/m^3^, a marked improvement compared to lower-strength, lighter-density samples without additives or with lower disintegration speeds (~1.09 MPa, 350 kg/m^3^).

Replacing PC with MOC binders provides rapid setting times and higher initial mechanical strength, facilitating shorter production cycles and earlier handling or installation. MOC-based panels exhibit excellent fire resistance and superior resistance to moisture, humidity, and mould, which prevent swelling, warping, or degradation in damp environments. MOC panels typically offer higher flexural and compressive strength than ordinary PC counterparts, enhancing structural resilience and impact resistance. These properties make MOC formulations particularly well suited for applications demanding durability under challenging environmental conditions.

The GCP binder leverages synergistic effects to enhance both mechanical performance and dimensional stability. The gypsum imparts rapid initial strength development, whereas pozzolan contributes to long-term strength gains by reacting with calcium hydroxide to produce additional cementitious compounds. This enhances water resistance and reduces degradation risks in wet environments. Experimental data have shown flexural strength increases of up to 48% and compressive strength improvements of up to 49% relative to two-component systems. Moreover, the system mitigates shrinkage and cracking, resulting in panels with superior dimensional stability and moisture-drought resistance ratios.

### 4.2. Improved Mechanical Resistance for Acoustic Panels Made with Wood Wool Filler and Alternative Binders

Improved mechanical resistance in acoustic panels made with wood wool filler is achieved primarily through alternative binders such as MOC and CAC. These binders provide several advantages over ordinary PC (Table 10).

### 4.3. Limitations and Suggestions for Further Optimisation and Industrial Scaling

Limitations and challenges for further optimisation and industrial scaling of acoustic panels with wood wool fillers and alternative binders are formulated in Table 11.

In the current study, the focus was primarily on establishing baseline flexural and compressive strengths of wood wool composites with various binder systems and additives, assessed in short-term mechanical tests. Although direct experimental data on creep, fatigue, or impact resistance are not included in this work, we discuss the potential behaviour based on the composite microstructure and binder characteristics. The dense interfacial bonding achieved through mechanical activation and aluminosilicate additives, as well as rapid curing and superior fire and moisture resistance of alternative binders such as MOC and CAC, suggests enhanced structural stability that could positively influence resistance to deformation under sustained or cyclic loads. Furthermore, improved dimensional stability and reduced shrinkage afforded by the ternary gypsum–PC–pozzolan (GCP) system may help mitigate creep-related issues.

To fully address these long-term mechanical performance aspects, we recommend and plan to conduct dedicated creep and fatigue testing protocols, as well as impact resistance evaluations, in future work tailored to suspended panel applications. Such studies will complement our current findings and support reliable industrial scaling.

To comprehensively validate the acoustic performance of the developed wood wool panels with novel mineral binders, future experimental investigations will focus on acquiring standardised sound absorption coefficients in accordance with ISO 354 (measurement of sound absorption in a reverberation room) [48] and ISO 11654 (rating of sound absorption) [49]. These standardised methods provide repeatable and comparable acoustic characterisation that is critical for benchmarking material performance against existing commercial acoustic panels and for certification purposes.

### 4.4. Suggestions for Optimisation and Scaling

Enhance Water Resistance: Incorporate mineral additives, salts, or modified MgCl_2_ activators (e.g., magnesium sulphate substitution) to reduce hygroscopicity and improve the moisture resistance of MOC-based panels. Composite Design: Explore fibre hybridisation and the use of pozzolanic additives to optimise the strength-to-weight ratio. Surface Treatments: Apply sol–gel coatings or similar methods to stabilise thermal and acoustic properties under humidity fluctuations.

### 4.5. Life Cycle Assessment Outcomes

The LCA analysis of acoustic boards presented in this report reveals significant variations in environmental impacts depending on the binder composition used. Among the evaluated options, the binder system consisting of GCP demonstrated a relatively low total carbon footprint, with total CO_2_ equivalent emissions around 5.644 kg per functional unit, indicating an efficient combination in terms of climate impact. Conversely, systems based on magnesium chloride binders with MgO exhibited notably higher emissions, reaching total CO_2_ equivalents of up to approximately 10.84 kg due to the significant contribution of MgO and MgCl_2_ in raw material emissions. The white PC binder systems showed moderate impacts, with total emissions around 6.19 kg CO_2_ eq, mainly driven by cement production. The incorporation of aluminosilicate additives, with or without a disintegrator, led to somewhat higher impacts than the pure white PC binder case, primarily due to the contributions of MK. Overall, the three-component GCP binder system offers the best balance of mechanical performance and minimised environmental impact, making it the preferred choice among the studied compositions for sustainable acoustic board production. Key raw material contributors to the environmental load are cement (various types), MgO, MgCl_2_, and MK, highlighting the importance of optimising binder formulations to reduce carbon emissions.

## 5. Conclusions

This study has demonstrated the successful development of a new eco-innovative mineral binder system designed to produce acoustic panels with a significantly reduced carbon footprint compared to ordinary PC-based materials. The key findings include the following:

- Incorporating aluminosilicate additives, particularly MK and liquid glass, combined with a high-speed disintegration process (5000 rpm), significantly enhances the mechanical properties of wood particleboard composites. Samples containing 20–30% metakaolin and liquid glass achieved peak flexural strengths up to 2.65 MPa and densities exceeding 490 kg/m3, representing substantial improvements over control samples without additives.
- Alternative binder systems, such as CAC and MOC, were successfully evaluated, offering advantages in rapid curing, superior fire resistance, and enhanced moisture and mould resistance. These properties contribute to improved durability and broader application potential in environments prone to moisture and fire.
- The ternary binder system comprising gypsum, PC, and pozzolan (GCP) exhibited synergistic effects, improving flexural and compressive strengths by up to 48% and 49%, respectively; reducing shrinkage and cracking; and enhancing dimensional stability and water resistance of the panels.
- Contrary to earlier assumptions, life cycle assessment results reveal that MOC and CAC formulations exhibit higher embodied carbon than ordinary PC-based systems, with CO2 equivalent emissions of approximately 10.84 and 7.94 kg/m2, respectively, compared to 5.40 kg CO2 eq./m2 for the reference PC panel. This increase is mainly due to the energy-intensive production processes of MgO, MgCl2, and CAC. These findings highlight the importance of carefully balancing enhanced mechanical and fire-resistant properties against environmental impacts when selecting alternative binders for sustainable wood wool panel production.
- Process parameters such as precise dosing of water, optimised mixing techniques, and controlled curing regimes were instrumental in producing homogeneous composite mixtures, ensuring batch consistency and reliable material performance.

## Figures and Tables

**Table 1:** Sizes of pine wood wool.

Length (mm)	Width (mm)
1.7 ± 0.15	0.16 ± 0.04

**Table 2:** PC CEM I 52.5 R composition.

Oxide	SiO_2_	Al_2_O_3_	Fe_2_O_3_	CaO	K_2_O	SO_3_	Na_2_O	MgO	Other
%	21.6	4.05	0.26	65.7	0.35	3.30	0.30	1.30	3.14

**Table 3:** Metakaolin composition.

Oxide	SiO_2_	Al_2_O_3_	Fe_2_O_3_	CaO	K_2_O	SO_3_	Na_2_O	MgO	Other
%	50.6	34.0	0.74	2.49	0.7	0.07	10.1	0.59	0.71

**Table 4:** CAC composition.

Oxide	Al_2_O_3_	Fe_2_O_3_	CaO	SiO_2_	Na_2_O + K_2_O	Other
%	69–71	<0.3	28–30	<0.5	<0.5	<0.2

**Table 5:** PC CEM I 42.5 R composition.

Oxide	SiO_2_	Al_2_O_3_	Fe_2_O_3_	CaO	K_2_O	SO_3_	Na_2_O	MgO	Other
%	18.8	5.23	3.34	62.98	0.88	3.11	0.51	1.49	3.66

**Table 6:** Key properties and environmental impact of selected binders.

Binder Type	Key Properties	Environmental Impact
Portland Cement	High mechanical strength, widely used, high CO_2_ emissions	High CO_2_ emissions
Metakaolin (Supplementary Cement Material)	Enhances strength, pozzolanic activity, and improves microstructure	Derived from clay or kaolin, reduces cement content
Liquid Glass (Additive)	Increases water resistance, densifies matrix	Low environmental impact
Calcium Aluminate Cement (CAC)	Rapid curing, sulphate and fire resistant, durable in moist environments	High CO_2_ emissions
Magnesium Oxychloride Cement (MOC)	Rapid setting, fire-resistant, moisture-resistant	Low carbon footprint, eco-friendly
Gypsum–Cement–Pozzolan (GCP)	Synergistic strength gain, reduced cracking, moisture, and sulphate resistance	Sustainable due to pozzolan use

**Table 7:** Composition of alternative binders.

Raw Material and Treatment	ASB Alumino-Silicate Binder	ASB-2 Alumino-Silicate Binder	ASB-5 Alumino-Silicate Binder	CACB Calcium Aluminate Binder	MOCB Magnesium Oxychloride Binder	GCP Binder System	Reference
Ordinary Portland cement (CEM I 42.5 R)							✓
White cement CEM I 52.5.R	✓	✓	✓			✓	
Metakaolin, 0, 5, 10, 20, and 30% of the cement	✓	✓	✓			✓	
Calcium aluminate cement				✓			
Magnesium oxide					✓		
Magnesium chloride					✓		
Gypsum						✓	
Desintegrator, 2000 rpm		✓					
Desintegrator, 5000 rpm			✓				
Water	✓	✓	✓	✓	✓	✓	✓
Liquid glass	✓	✓	✓	✓		✓	✓

**Table 8:** Physical–mechanical properties of wood wool composite samples with a thickness of 25 mm.

Property	Bulk Density (kg/m^3^)	Compressive Strength 10% Strain (MPa)	Flexural Strength (MPa)	Thermal Conductivity (W/(m·K))	Thermal Resistance (m^2^·K/W)
Reference	420	≥0.3	≥1.3	0.066	0.35
WPC White Portland Cement	381.1–503.2	0.28–0.51	1.31–2.00	0.068–0.074	0.33–0.34
ASB Aluminosilicate without Disintegrator	411.1–519.9	0.26–0.60	1.09–2.65	0.069–0.71	0.35–0.36
ASB-2 Aluminosilicate with Disintegrator, 2000 rpm	351.0–466.7	0.34–0.56	1.67–2.13	0.066–0.073	0.33–0.35
ASB-5 Aluminosilicate with Disintegrator, 5000 rpm	403.9–417.2	0.46–0.57	171–1.86	0.066–0.068	0.34–0.35
CACB Calcium Aluminate Cement	368.4–430.6	0.23–0.40	1.66–2.58	0.074–0.076	0.33–0.34
MOCB Magnesium Chloride	380.3–442.6	0.33–0.36	1.24–1.84	0.081–0.084	0.30–0.31
GCP System	396.9–409.9	0.32–0.45	1.35–1.87	0.067–0.069	0.36–0.37

**Table 9:** Physical–mechanical properties of optimised wood wool samples with a thickness of 25 mm.

Property	Benchmark	White Portland Cement	Aluminosilicate w/Disintegrator (ASB-5)	Calcium Aluminate Cement (CACB)	Magnesium Chloride (MOCB)	GCP System
Material density (kg/m^3^)	420	483.69	423.22	400.73	389.49	416
Weight (kg/m^2^)	10.5	12.09	10.58	10.02	9.74	10.4
Thermal Resistance (m^2^·K/W)	0.35	0.34	0.37	0.34	0.31	0.37
Thermal Conductivity (W/m·K)	0.066	0.073	0.068	0.074	0.081	0.068
Flexural Strength (MPa)	≥1.3	1.70	2.24	2.00	1.84	1.6
Compressive Strength 10% Strain (MPa)	≥0.3	0.44	0.46	0.31	0.36	0.6
Modulus of Elasticity (N/mm^2^), k = 2000	2600	3400	4480	4000	3680	3200

**Table 10:** Distinctive advantages of using MOC and CAC over PC.

Distinctive Feature	Description of MOC/CAC Advantages
Enhanced Strength and Durability	MOC exhibits excellent adhesion to organic fillers like wood wool, leading to the composites’ superior flexural and compressive strengths. This results in improved impact resistance and structural performance of the panels. CAC offers high early strength and resistance to extreme conditions like high temperatures and chemical exposure, further enhancing wood wool composites’ durability and mechanical resistance. Panels with magnesium chloride binders (including MOC) show excellent fire, moisture, and mould resistance, which contribute to maintaining mechanical integrity over time, especially in humid environments.
Rapid Curing and Production Efficiency	The rapid-setting nature of MOC facilitates faster curing, enabling reduced production cycle times and earlier handling or installation without compromising mechanical properties. This rapid setting also benefits prefabricated panel manufacturing, improving construction efficiency and reducing labour costs.
Optimisation of Composite Structure	The wood wool-to-binder ratio (typically between 0.43 and 0.57) influences mechanical resistance, which balances panel density (300–550 kg/m^3^) and strength. Surface treatments and hybridisation strategies enhance the interfacial bonding between wood fibres and the binder, improving stress transfer and resistance to cracking.
Moisture Resistance and Hygroscopicity Management	MOC panels resist swelling, warping, and mould growth in moist conditions, which are critical for preserving mechanical performance in wet environments such as bathrooms and outdoor applications. Mineral additives, salts, and substitution of magnesium chloride solution with magnesium sulphate have been shown to reduce the hygroscopicity of MOC, further stabilising mechanical properties under variable humidity.

**Table 11:** Limitations and challenges for further optimisation of wood wool panel.

Limitations and Challenges	Description	Envisaged Solution
Moisture Sensitivity and Hygroscopicity	Magnesium oxychloride cement (MOC), despite excellent adhesion and mechanical strength, has relatively low water resistance and high hygroscopicity, which limit its application in environments with high relative humidity or outdoor exposure. Moisture-related durability issues, such as swelling, warping, and mould growth, remain concerns that require mitigation.	Applying sol–gel coatings or similar surface treatments can stabilise thermal and acoustic performance under varying humidity conditions by providing a protective barrier against moisture ingress.
Durability Under Environmental Stress	Panels must be tested and optimised for long-term durability, including freeze–thaw resistance, fire resistance, and resistance to biogenic sulphur corrosion to ensure structural integrity and performance.	Inclusion of pozzolanic additives and fibre hybridisation may improve mechanical strength and dimensional stability, thus enhancing resistance to environmental degradation, such as cracking or shrinkage induced by moisture fluctuations.
Balancing between Strength and Density	Achieving the optimal balance between lightweight characteristics (200–600 kg/m^3^) and mechanical strength is critical. Excessive density increases weight and cost, while low density may reduce strength and acoustic performance. Further fibre hybridisation and inclusion of pozzolanic additives can be explored to improve this balance.	Fine-tuning of wood wool-to-binder ratio between 0.43 and 0.57 allows for balancing panel density (300–550 kg/m^3^) and mechanical resistance, achieving an optimal compromise between weight and performance.
Heat and Moisture Stability	Thermal conductivity and acoustic performance can be affected by hygroscopicity. Stabilising these properties under varying humidity using sol–gel coatings or other surface treatments is an area for improvement.	Utilising rapid-setting binders like CAC and MOC allows for shorter processing cycles and improved initial strength, contributing to better stability against thermal- and moisture-induced deformation.
Material Variability and Quality Control	Variability in raw bio-fibre and wood materials can lead to inconsistent panel properties, posing challenges for standardisation and mass production. For industrial scaling, reliable and reproducible panel manufacturing processes are needed that ensure homogeneous binder distribution and panel compaction.	Employing high-speed disintegration (up to 5000 rpm) during composite preparation to enhance particle comminution and structural uniformity improves interfacial bonding and consistency in mechanical properties.

## Data Availability

The data presented in this study are available on request from the corresponding author due to the potential commercial use of the development.

## Abbreviations

The following abbreviations are used in this manuscript:

CA/CACB	Calcium aluminate cement
MOC/MOCB	Magnesium oxychloride cement
GCP	Gypsum, cement, and pozzolan composition
ASB	Aluminosilicate without desintegrator
ASB-2	Aluminosilicate with desintegrator, 2000 rpm
ASB-5	Aluminosilicate with desintegrator, 5000 rpm
